# Morphological and functional abnormalities of the orofacial sphere associated with thumb sucking in children aged 3 to 10 years old in Yaounde, Cameroon

**DOI:** 10.11604/pamj.2022.42.107.33050

**Published:** 2022-06-09

**Authors:** Ginette Claude Mireille Kalla, Eunice Danielle Medou Tiomo, Jules Onana, Francois-Xavier Mbopi-Keou, Charles Bengondo Messanga

**Affiliations:** 1 Faculty of Medicine and Biomedical Sciences, University of Yaounde I, Yaounde, Cameroon,; 2 Pediatric Unit, Yaounde University Teaching Hospital, Yaounde, Cameroon,; 3 Yaounde Military Hospital, Yaounde, Cameroon,; 4 ICT University, Yaounde, Cameroon,; 5 Stomatology Unit, Yaounde University Teaching Hospital, Yaounde, Cameroon

**Keywords:** Morphological abnormalities, functional abnormalities, orofacial sphere, thumb sucking, children, Yaounde, Cameroon

## Abstract

**Introduction:**

thumb sucking is one of the most common oral habits in infants and children. In our context, little is known about the effects of prolonged thumb sucking on the orofacial sphere. Objective: determine the prevalence of thumb sucking and, identify the morphologic and functional abnormalities of the orofacial sphere associated with the duration and frequency of thumb sucking in children aged 3 to 10 years.

**Methods:**

a cross-sectional and analytical study was carried out in the pediatric units of the District Hospitals of Biyem-assi and Efoulan from February to June 2020 in children aged 3 to 10 years. Recruitment was consecutive, not probabilistic. After obtaining informed parental consent, a clinical examination was performed and the criteria retained were based on the ANGLE classification of the malocclusions. Socio-demographic, socio-economic, clinical characteristics were collected and morphological and functional abnormalities were observed. Statistical analysis was performed using SPSS software version 23.0. The significance threshold was set at 5%.

**Results:**

of the 116 enrolled children; 74 girls (63.79%) and 42 boys (36.21%) with a mean age of 4.80 ± 0.5 years. The highest proportion of thumb sucking was found in firstborn children (n=46, 39.65%), and in those who breastfed for less than 6 months (n=99, 85.62%). The prevalence of thumb sucking was 17.4%. Using the multivariate analysis, class II division 1 canine occlusion [OR=1.52 (1.27-2.68), p=0.03] and decreased overbite [OR=4.5 (2.5-9.3), p=0.001] while class II division 1 canine occlusion [OR=2.59 (1.3-10.1), p=0.009] and increased overjet [OR=1.89 (1.06-6.75), p=0.005] were independent morphologic abnormalities significantly associated with the frequency and the duration of thumb sucking respectively. There was no association between the duration and frequency of thumb sucking and the functional abnormalities.

**Conclusion:**

thumb sucking is more common in girls and the likelihood of thumb sucking decreases with age. The prevalence of thumb sucking was 17.4%. The malocclusions observed in our population are class II division 1 canine relationship, decreased overbite and increased overjet.

## Introduction

The orofacial sphere, is a muscular and skeletal apparatus of the head and neck, characterized by close functional and morphological connections. An incorrect function or morphology of this apparatus may lead to abnormalities in the orofacial area, reversible at first thanks to well-conducted prophylaxis [1]. At birth, sucking is a natural reflex for newborns as it enables the child to breastfeed [2]. However, it can be engrained in a habit that is very hard to break for some children [3]. Thumb-sucking has been described as a common childhood habit that is considered normal up to the age of 3 to 4 years old [3,4]. Deleterious muscle behaviors due to sucking are often associated with impeded osseous growth, tooth malposition, disturbed breathing habits, difficulties in speech, upset balance in the facial musculature and psychological problems [5-7].

The etiology behind the initiation of the thumb sucking habit among children has been extensively investigated and divergent explanations have appeared in the literature [2,8]. Some of these include: behavioral and mood changes, reactions to family differences and lack of affection [2,9]. The increased use of pacifiers in some civilized countries of the world has resulted in marked reduction in the prevalence of thumb sucking [7,10]. The prevalence is reported to be between 13 and 100% in some societies [11]. Kerosuo, reported the prevalence of sucking habit on an African group (Tanzanians) as 10% while 4 and 10% for Asian/Arab and Finnish children respectively [12]. Oral habits such as thumb sucking are among the most evident etiology of malocclusion. It is well accepted that non nutritive sucking habit persisting beyond 3 years of age are implicated in the development of anterior open bite [13].

Thereby, there is increasing emphasis on early detection of conditions predisposing young children to malocclusion around the world, and preventive and interceptive procedures are increasingly being implemented [4,10]. It therefore seems essential to focus on this important aspect of modern orthodontics in our context. Indeed, there is little or no information concerning the effects of thumb sucking upon the occlusion of the children in Cameroon. This is why, we decided to undertake this study with the aim to determine the prevalence of thumb sucking and, identify the morphologic and functional abnormalities of the orofacial sphere associated with the duration and frequency of thumb sucking in children aged 3 to 10 years.

## Methods

**Type and place of study:** an analytic cross-sectional study was carried out for a period of 5 months from February to June 2020 at the pediatric unit of the Biyem-Assi and Efoulan District Hospitals.

**Duration and period of study:** the duration was 10 months from November 2019 to August 2020. Data was collected for a period of 5 months from 4^th^ February 2020 to 29^th^ June 2020.

**Study population:** children aged 3 to 10 years involved in thumb sucking and who their parents had given their consent were included. Those with para-functional habits (such as tongue sucking, pacifier use, nail biting) that can lead to disorders similar to those induced by thumb sucking on the orofacial sphere and those with extensive caries were excluded. Based on the prevalence of digit-sucking of 8.1% [10], the calculated sample size was 114 children. On the six hundred and eighty-nine children aged from 3 to 10 years that visited the above Hospitals during our study period, 120 were involved in thumb sucking. Of these 120 children who were eligible, four were excluded because of the non-consent of their parents. The study ultimately focused on 116 children.

**Procedure:** after obtaining the informed consent of the parents, we did a physical examination of these 116 children and the criteria applied to assess them were based on ANGLE´s classification of mal occlusions [14]. The overjet was measured from the palatal surface of the most protruded fully erupted maxillary incisor to the labial surface of the corresponding mandibular incisor in centric occlusion. This distance was measured in millimeters (mm) using a metal millimeter ruler. This distance is normally 2 to 3 mm [15]. In our study, a value greater than 3.5 mm was considered an increased overjet. The anterior openbite was recorded when there were no vertical contacts between the upper and lower incisal edges [16]. The class 2 division 1 canine relationship was recorded when the tip of the maxillary primary canine tooth was mesial to the distal surface of the mandibular primary canine and when the lower incisor edges lie posterior to the cingulum plateau of the upper incisor [17].

**Study variables:** the variables studied were socio-demographic (age and sex), socio-economic (mother´s occupation, socio-economic status of the family) and clinical variables (birth rank, breastfeeding duration, duration (< 1 year or > 1 year) and frequency (< 6 hours/day or > 6 hours/day) of thumb sucking) as well as the morphological (Class II division 1 canine relationship, increased overjet, anterior open-bite) and functional (atypical infantile swallowing) outcome induced by thumb sucking on the orofacial sphere. The socio-economic status of the family was according to the minimal interprofessional guarantee salary.

**Data management and analysis:** we obtained data using a pre-established questionnaire. Data was entered in a data entry application CSPro 7.1.0, and then exported to SPSS version 23.0. Categorical variables were presented as frequencies and percentages. Continuous variables were presented as means (and standard deviation) or median (and 25^th^ and 75^th^ inter-quartile range) where appropriate. The strength of association was assessed using the odds ratio and the confidence interval at 95%. The level of statistical significance was set at a p-value of less than 0.05. In order to exclude any confounding factor, multivariate analysis was made on a logistic regression model.

**Ethical and administrative procedures:** the study was approved by the Institutional Ethical Review Committee of the Faculty of Medicine and Biomedical Sciences, University of Yaounde I, and the administrative authorizations from the directors of the Biyem-assi and the Efoulan District Hospitals were obtained. Voluntary informed consent was obtained from the parents. Our study did not interfere with the physical integrity of the patients nor their management, and anonymity was maintained throughout the study.

## Results

During our study period, 120 children responding to our inclusion criteria were identified. Four were excluded because of the non-consent of their parents. The study ultimately focused on 116 children.

**Prevalence of thumb sucking:** six hundred and eighty-nine children of 3 to 10 years old attending the Biyem-assi and Efoulan District Hospitals were registered. One hundred and twenty were eligible giving us a prevalence of 17.4%.

**Description of the study population:** the mean age of the study population was 4.80±0.5 years with a minimum of 3 and a maximum of 10 years old. The most representative age group was children of 3 to 5 years old (n=66, 56.9%). The female gender was the most represented (n=74) with a percentage of 63.8% giving a sex ratio of 0.57. Children who came from a low socioeconomic background were the most represented (n=50, 43.1%) and those with working mothers (n=60) with a percentage of 51.72%. First born children were the most represented to indulge in thumb sucking (n=46, 39.65%). The most predominant thumb suckers were those who breastfed for less than 6 months (n=85, 73.27%). Concerning the duration and frequency of the habit, those who sucked daily for more than a year were the most represented with 57.76% and 81.90% respectively (Table 1). Children involved in thumb sucking had short and hypotonic upper lips (n=67, 57.75%), hypertonic lower lips (n=67, 57.75%), clearer and calloused thumb (n=94, 81.03%), and lowered tongue position (n=104) with a percentage of 89.65%. In the sagittal plane, they exhibited a class II incisor relationship (n=63, 54.31%), class II canine relationship (n=63, 54.31%), overjet ≥3.5mm (n=70, 60.34%). In the vertical plane, they had a decreased overbite (n=51, 43.97%) and in the transverse plane, they had a posterior cross bite (n=8, 6.9%). Most of the children involved in thumb sucking developed an atypical infantile swallowing (n=78) with a percentage of 67.24%, a normal mastication and respiratory pattern with a percentage of 85.34% and 76.72% respectively (Table 2, Table 3).

**Table 1:** distribution of the sample according to the sociodemographic, socioeconomic and clinical characteristics

Variables	Effective(n)	Percentages (%)
**Ages(years)**		
[3-5]	66	56.9
[5-7]	42	36.2
[7-9]	7	6.03
[9-10]	1	0.87
**Gender**		
Masculine	42	36.2
Feminine	74	63.8
**Socioeconomic status**		
Low	50	43.10
Medium	45	38.79
High	21	18.11
**Mother's occupation**		
Working mother's	60	51.72
Non-working mother's	56	48.28
**Birth rank**		
First child	46	39.65
Second child	33	28.44
Third child	20	17.24
Fourth child	17	14.65
**Breastfeeding duration**		
<6 months	85	73.27
≥ 6months	31	26.73
**Frequency of thumb sucking**		
<6 Hours/day		
≥6 Hours/day	49	42.24
**Duration of thumb sucking**	67	57.76
<1 year	21	18.10
≥1 year	95	81.90
**Total**	116	100

**Table 2:** sample distribution according to the morphological disorders

Variables	Effectives (n)	Percentage (%)
**Upper lip function**		
Short and hypotonic	67	57.75
Long and hypertonic	49	42.25
**Lower lip function**		
Hypotonic	49	42.25
Hypertonic	67	57.75
**Aspect of thumb**		
Clearer and calloused	94	81.03
Normal	22	18.97
Tongue placement		
Lowered tongue position	104	89.65
Lies freely	12	10.35
**Sagittal plane incisal relationship**		
Class I	52	44.83
Class II	63	54.31
Class III	1	0.86
**Canine relationship**		
Class I	52	44.83
Class II	63	54.31
Class III	1	0.86
**Overjet**		
<3.5mm	46	39.66
≥3.5mm	70	60.34
**Vertical dimension**		
Normal	27	23.27
Open-bite	38	32.76
Overbite	51	43.97
**Transverse dimension**		
Normal	108	93.10
Posterior cross bite	8	6.90
**Total**	116	100

**Table 3:** sample distribution according to the functional abnormalities

Variables	Effectives (n)	Percentages (%)
**Mastication**		
Normal	99	85.34
Painful/ atypical	17	14.66
**Deglutition**		
Normal	38	32.76
Infantile	78	67.24
**Respiration**		
Normal	89	76.72
Mouth breathing	27	23.28
**Total**	116	100

**Bivariate analysis of the frequency of thumb sucking according to morphological abnormalities:** there was a significant association between the frequency of thumb sucking and the class II incisal relationship [OR=4.84 (1.69-13.8), p value=0.002], Class II canine relationship [OR=3.40 (1.24-9.29), p value=0.014], decreased overbite [OR=2.40 (1.15-9.12), p value=0.004] and the overjet > 3.5 mm [OR=2.87 (1.27-15.4), p value= 0.003] (Table 4).

**Table 4:** association between the frequency of thumb sucking and the morphological disorders

Variables	Frequency of thumb sucking, n (%)		OR (CI at 95%)	P-value
Upper lip function	<6 hours/day	>6 hours/day		
Long and hypertonic	38(44.19)	16(55)	0.65(0.24-1.72)	0.38
Short and hypotonic	48(55.81)	14(45)	1.54(0.58-4.11)	
**Lower lip function**				
Hypotonic	38(44.19)	16(55)	0.65(0.24-1.72)	0.38
Hypertonic	48(55.81)	14(45)	1.54(0.58-4.11)	
**Aspect of the thumb**				
Clearer and calloused	78(90.7)	22(85)	0.58(0.14-2.42)	0.45
Normal	8(9.3)	8(15)	1.72(0.41-7.17)	
**Sagittal plane**				
**Incisor relationship**				
Class I	42(65.31)	7(28)	0.49(0.06-3.70)	
Class II	17(34.69)	18(72)	4.84(1.69-13.8)	0.002
**Canine relationship**				
Class I	34(69.39)	10(40)		
Class II	15(30.61)	15(60)	3.40(1.24-9.29)	0.014
**Overjet**				
<3.5mm	34(69.39)	7(28)	0.49(0.06-3.7)	
>3.5mm	15(30.61)	18(72)	2.87(1.27-15.4)	0.003
**Vertical plane**				
Normal	11(22.45)	0(00)		
Decreased overbite	12(24.49)	17(68)	2.40(1.15-9.12)	0.004
Open-bite	26(53.06)	8(32)		
**Transverse plane**				
Normal	80(93.02)	19(95)	1.43(0.16-12.5)	
Posterior cross bite	7(6.98)	1(5)	0.70(0.08-6.18)	0.83

**Bivariate analysis of the duration of thumb sucking according to morphological abnormalities:** the short and hypotonic upper lip function [OR=5.96 (2-17.73), p value=0.041], hypertonic lower lip function [OR=5.96 (2-17.73), p value=0.041], the development of a class II incisor relationship [OR=2.93 (0.78-10.6), p value=0.001], class II canine relationship [OR=3.48(1.24-9.29), p value=0.005], overjet > 3.5mm [OR=3.60(1.42-10.9), p value=0.01] and a decreased overbite [OR=3.17(1.28-9.08), p value=0.002] were significantly associated with the duration of thumb sucking (Table 5).

**Table 5:** association between the duration of thumb sucking and the morphological disorders

Variables	Duration of thumb sucking, n (%)		OR (CI at 95%)	P-value
Upper lip function	<1 year	>1 year		
Short and hypotonic	32(65.31)	6(24)	5.96(2-17.73)	0.041
Long and hypertonic	17(34.69)	19(76)	0.17(0.06-0.5)	
**Lower lip function**				
Hypotonic	17(34.69)	19(76)	0.17(0.06-0.5)	
Hypertonic	32(65.31)	6(24)	5.96(2-17.73)	0.041
**Aspect of the thumb**				
Clearer and calloused	47(95.92)	23(92)	2.04(0.27-15.4)	0.48
Normal	2(4.08)	2(8)	0.49(0.06-3.7)	
**Sagittal plane**				
**Incisor relationship**				
Class I	10(50)	32(37.21)	0.35(0.09-1.28)	
Class II	10(50)	54(62.79)	2.93(1.78-10.6)	0.001
**Canine relationship**				
Class I	15(75)	57(66.28)	0.21(0.07-0.59)	
Class II	5(25)	29(33.72)	3.48(1.24-9.29)	0.005
**Overjet**				
<3.5mm	34(69)	2(8)		
>3.5mm	32(65.31)	23(92)	3.60(1.42-10.9)	0.01
**Vertical plane**			0.14(0.05-0.41)	
Normal	2(10)	13(15.12)		
Open-bite	6(30)	32(39.54)		
Decreased overbite	12(60)	39(45.35)	3.17(1.28-9.08)	0.002
**Transverse plane**				
Normal	52(61.9)	32(85)	1.33(0.5-3.56)	
Posterior cross bite	1(38.1)	7(15)	0.75(0.28-2.0)	0.65

**Bivariate analysis of the frequency of thumb sucking according to functional abnormalities:** no significant association was found between the frequency of thumb sucking and the functional abnormalities.

**Bivariate analysis of the duration of thumb sucking according to functional abnormalities:** infantile deglutition was significantly associated to the duration of thumb sucking with [OR=6.11(1.28-29.08), p value=0.0018] (Table 6).

**Table 6:** association between the duration of thumb sucking and the functional defects

Variables	Duration of thumb sucking, n (%)		OR (CI at 95%)	P-value
Mastication	< 1 year	>1 year		
Normal	37(77.08)	20(72)	1.31(0.43-3.94)	
Painful/atypical	11(22.92)	7(28)	0.76(0.25-2.3)	0.63
**Deglutition**				
Normal	36(73.47)	7(28)	0.16(0.03-0.78)	
Infantile	13(26.53)	18(72)	6.11(1.28-29.08)	0.0018
**Respiration**				
Normal	68(79.07)	8(40)	0.42(0.09-1.98)	
Mouth breathing	18(20.93)	12(60)	2.69(0.51-11.23)	0.106

**Multivariate analysis of morphological abnormalities of the orofacial sphere associated with the frequency of thumb sucking:** independent factors significantly associated to the frequency of thumb sucking were the development of a class II incisor relationship (p=0.03), class II canine relationship (p=0.007) and a decreased overbite (p=0.001).

**Multivariate analysis of morphological abnormalities of the orofacial sphere associated with the duration of thumb sucking:** independent factors significantly associated to the duration of thumb sucking were the development of a class II incisor (p=0.009), canine relationship (p=0.013) and an increased overjet (p=0.005).

**Multivariate analysis of functional abnormalities of the orofacial sphere associated with the frequency and duration of thumb sucking:** no independent factors significantly associated were found.

## Discussion

The oral habits of the child constitute one of the main etiological factors leading to anomalies of the orofacial sphere in the world [4,7,18]. In this study we sought to assess the effects of the duration and frequency of thumb sucking on the orofacial structures in children of 3 to 10years old of our population.

**Prevalence of thumb sucking:** the prevalence of sucking habits varies between different countries [19]. Scandinavian studies report the frequency of sucking habits to be slightly above 80%, with dummy sucking as the predominant type. In contrast to digit (finger or thumb) sucking, the use of pacifiers decreased considerably during the pre-school period and at the age of four the majority of children had given up their dummy sucking habit [4]. A Nigerian study on a group of children demonstrated that 13.1% developed a habit in early life [20]. In our country we obtained a prevalence of thumb sucking habit of 17.4%. This result is similar to Ibrahim Ngom *et al*. [21] in Senegal in 2008 and Quashie R *et al*. [22] in Nigeria in 2010 that reported a prevalence of 16.5% and 17% respectively but differs to the report of Dhull S *et al*. [23] who found a prevalence of 12.8% in India in 2018.

**Socio-demographic and socio-economic characteristics:** we observed that the mean age of our study population was 4.80±0.5 years and that the percentage of children with the habit decreased as age increases. This can be explained by the fact that when entering school, there are mocked by their peers and turn to leave the habit more quickly. This result agrees with previous studies which show a decrease percentage of the habit with age [4,20,24]. The girls are more involved in the habit than boys. This result agrees with previous studies which suggest that girls demonstrated a higher level of sucking habits than boys [25,26], but it differs with other studies who showed that boys exhibit the habit more than girls [27]. This can be explained by the fact that girls are more reserved, they are inclined to be less active and inclined to fatigue. Generally, they show no special attachment either toward bottle or breastmilk.

**Clinical characteristics:** in the literature, an increased duration of breastfeeding has often been associated with a reduced incidence of malocclusion [24]. This is in accordance with our study which showed a higher percentage of thumb suckers in children who breastfed for more than six months. This can be explained by the fact that breastfeeding stimulates normal craniofacial growth and development and prevents the child from indulging in non-nutritive sucking habits. Clinical experience suggests that, in order to move teeth, a force must be applied for at least six hours per day [28]. This is accordance with our study which showed that children who sucked for more than 6 hours daily were more represented and had the higher disorders.

**Morphological disorders:** a significant relationship between thumb sucking habit and the observed occlusal discrepancies in this study population is in line with the previous epidemiological reports [1,6,7]. In the sagittal dimension, the results of this study agree with previous studies in that thumb sucking is associated with a class II division 1 canine relationship and also increased overjet [29]. The higher incidence of increased overjet may be due to proclination of the maxillary incisors and forward displacement of the maxillary base as a result of the pressure of the thumb [7,11]. Johnson *et al*. observed that thumb sucking habit led to abnormal pressure in both upper and lower maxilla and the teeth, the lower teeth act as a fulcrum and the teeth are returned [14]. The overjet may also be worsened by retroclination of the lower incisors due to the lever action of the thumb [7]. The increase in class II canine relationships may be due to the forward displacement of the anterior maxillary base [7].

In our study, thumb sucking was associated with an increased overjet and decreased overbite. This was also found by many studies [11,28,29]. In the vertical dimension, the results of this study agree with those of existing studies in that thumb sucking is associated with an increased open bite [30]. In his study, his results revealed that sucking habits of 1-2 hours and duration of 24 to 60 months exhibited the highest prevalence of an anterior open bite in 13 (44.8%) and 17 (58.6%) children respectively [30]. Indeed, pressure from the thumb hinders the downward growth of the maxillary base and delays the anterior teeth from erupting while the posterior teeth continue to erupt. This results in over eruption of the posterior teeth and the formation of an anterior open bite. Moimaz *et al*. also found that, children with a finger sucking habit, as well as those with low rates of breastfeeding, were more susceptible to overjet and open bite [31]. There was no association between thumb sucking habits in the transverse dimension. These inconsistent findings can be explained by the fact that most of the studies have not accounted for confounding factors, such as age, gender in their statistical analyses.

**Functional disorders:** in this study we found that the duration of thumb sucking was significantly associated to infantile swallowing, but there is no relationship with the masticatory and respiratory pattern. Kasparaviciene *et al*. in 2014 found infantile swallowing in 5.4% of the children in their sample and in their study, this type of infantile swallowing was associated with anterior open bite (p = 0.001) [13]. Using the multivariate analysis, the functional discrepancies examined were neither associated with the frequency nor the duration of thumb sucking.

**Study limitation:** the COVID-19 pandemic disease made our collect difficult due to the few patients who visited our hospitals.

## Conclusion

The results of our study enable us to make the following conclusions: (i) the prevalence of thumb sucking in children of 3 to 10 years old in our country is 17.4%; (ii) class II division 1 canine occlusion and decreased overbite while class II division 1 canine occlusion and increased overjet were independent morphologic abnormalities significantly associated with the frequency and the duration of thumb sucking respectively; (iii) there was no association between the duration and frequency of thumb sucking and the functional abnormalities; (iv) this work shows the interest of detecting and interrupting this habit sufficiently early, with the aim of avoiding the above-mentioned abnormalities.

### What is known about this topic

- The orofacial sphere, is a muscular and skeletal apparatus of the head and neck, characterized by close functional and morphological connections; an incorrect function or morphology of this apparatus may lead to abnormalities in the orofacial area, reversible at first thanks to well-conducted prophylaxis.

### What this study adds

- The prevalence of thumb sucking in children of 3 to 10 years old in our country is 17.4%;
- Class II division 1 canine occlusion and decreased overbite while class II division 1 canine occlusion and increased overjet were independent morphologic abnormalities significantly associated with the frequency and the duration of thumb sucking respectively;
- There was no association between the duration and frequency of thumb sucking and the functional abnormalities; this work shows the interest of detecting and interrupting this habit sufficiently early, with the aim of avoiding the above-mentioned abnormalities.
